# Multiple mutations and overexpression of the *MdaE7* carboxylesterase gene associated with male-linked malathion resistance in housefly, *Musca domestica* (Diptera: Muscidae)

**DOI:** 10.1038/s41598-017-17325-x

**Published:** 2018-01-09

**Authors:** Yi Zhang, Jing Li, Zhuo Ma, Chao Shan, Xiwu Gao

**Affiliations:** 1 Department of Entomology, China Agricultural University, Beijing, 100193 P.R. China; 2 Beijing Center for Diseases Prevention and Control, Beijing, 100013 China

## Abstract

Two unique housefly strains, MSS and N-MRS, were selected and used to clarify mechanisms of sex-associated malathion resistance in the housefly, *Musca domestica*. Compared with the lab-susceptible CSS strain, susceptible females and resistant males were observed in the malathion-susceptible MSS strain, while the malathion-resistant near-isogenic line, N-MRS, achieved similar resistance level between genders. Significant synergistic effect of the esterase-inhibitor DEF on resistant houseflies pointed to the important involvement of esterase in this specific malathion resistance. Examination of the carboxylesterase gene *MdαE7* in malathion resistant housefly populations found seven, non-synonymous SNP mutations (Ser^250^-Thr, Trp^251^-Ser, Met^303^-Ile, Leu^354^-Phe, Ser^357^-Leu, Trp^378^-Arg and Ser^383^-Thr), not found in susceptible houseflies, revealing a strong correlation between these mutations and the development of malathion resistance. Further genetic analysis conducted with bioassays by topical application and nucleotide polymorphism detection provided a first line of molecular evidence for a linkage between a male-determining factor and *MdαE7* gene in the MSS and N-MRS males. This linkage results in a much higher level of malathion resistance for males than females in the MSS strain. Lastly, quantitative real-time PCR showed that *MdαE7* was over expressed in the resistant strain due to the increased transcription level of mRNA rather than gene duplication.

## Introduction

The housefly, *Musca domestica* L. is a serious threat to human and animal health by carrying more than 100 pathogens^1^. Insecticides have been utilized for decades as the main tool to control houseflies. Malathion, an organophosphate (OP) insecticide, is widely used against houseflies owing to its toxicity profile^2^. Unfortunately, resistance to malathion in housefly was detected since the 1960s^3^.

Esterase-mediated resistance has been identified as one of the main mechanisms in OP resistance among insects^4^. In several dipteran and hemipteran insects, it is generally accepted that OP resistance is mainly mediated by enhanced metabolic detoxification or sequestration through quantitative changes in activity of general esterases^5^. Quantitative changes, such as gene duplication or up-regulation, or a combination of both, lead to the overproduction of enzymes^6^. The most comprehensive research on insecticide detoxification by gene amplification involved the overexpression of a specific carboxylesterase in *Myzus persicae*. This study demonstrated that the duplication of the genes E4, or its paralog FE4, were responsible for enhanced degradation and sequestration of insecticides^7^. Studies on other species, for example *Culex pipiens* (Diptera: Culicidae)^8^ and *Nilaparvata lugens* (Hemiptera: Delphacidae)^9^, provided more evidence for this resistance mechanism. Additionally, the up-regulation and elevated expression of esterase-encoding genes were observed in many species, such as B-biotype of *Bemisia tabaci* (Hemiptera: Aleyrodidae)^10^ and *Aphis gossypii* (Glover) (Hemiptera: Aphididae)^11^.

Structural changes in carboxylesterase, which confer an enhanced ability to metabolize the insecticide, are considered to be another important contributor in esterase-based OP resistance. In dipteran insects, a malathion-specific carboxylesterase (MCE), which hydrolyzes the insecticide to the less toxic and more easily excreted monoacids, has been implicated to be involved in resistance to malathion^12^. Research by van Asperen and Oppenoorth found that resistance to OP in housefly was always accompanied by reduced activity of ali-esterase in several resistant strains^13^. This relationship was explained by the so-called ‘mutant ali-esterase theory’, which proposed that the reduced ability to hydrolyze carboxylesterase substrates associated with an acquired ability to hydrolyze organophosphate substrates was caused by structural mutations in carboxylesterase^14,15^. Furthermore, mutational studies on OP resistance provided powerful, molecular evidence for this theory. *LcαE7* and *MdαE7*, a pair of homologous genes encoding carboxylesterase E3 in *Lucilia cuprina* and *Musca domestica*, respectively, were identified to be associated with both the loss of E3′s carboxylesterase activity and the acquisition of a novel OP hydrolysis activity. The same mutation Gly^137^Asp in both genes was proposed to be responsible for diazinon resistance, while another substitution Trp^251^Leu in *LcαE7* and Trp^251^Ser in *MdαE7* was involved in malathion resistance^2,16,17^. Since these seminal studies, mutation of esterase genes mediating OP resistance has been identified in many insect species^18^.

In houseflies of the so-called “standard” strains where females carry XX and males carry XY sex chromosomes sex is determined by a male-determining factor (M factor) located on the Y chromosome that blocks the female-determining factor (F factor)^19^. However, in many populations, the heterozygous or homozygous presence of the M factor has been discovered on one or multiple autosomes, or even rarely on the X chromosome^20,21^. Due to the presence of multiple M factors in the same individual, the F factor has evolved a dominant, constitutive mutation, *F* ^*D*^, to trigger female development^19^. The first case of sex-associated insecticide resistance in housefly, reported in 1990, showed females were more resistant against permethrin than males^22^. Later studies focusing on malathion as well as spinosad resistance indicated that the correlation of insecticide resistance to sex in housefly was not coincidental^23,24^. Interestingly, unlike the other two insecticides, the higher level of malathion resistance was observed in males rather than females. Kerr reported a segregation linkage between an autosomal M factor and a DDT resistance gene through research into the genetic basis of DDT resistance^25^. Moreover, similar conclusions were found in both pyrethroids and OP resistance^22,23^. Due to the finding that M factors can be present in different linkage groups in diverse housefly populations, the likelihood of an association between the M factor and a transposable genetic element was proposed^26^. Subsequent genetic analysis showing the M factor moved to chromosome 2 from chromosome 3 as a consequence of selection with malathion supported this suggestion^23^. Recently, the M factor has been identified to be originated from a duplication of a spliceosomal factor gene^27^. However, the molecular basis of this linkage is still unclear despite two decades of research.

Near-isogenic lines (NILs) refer to inbred populations of organisms with an approximately identical genetic background to the recurrent parent, except for loci chromosomally close to the target genotype. Although the susceptible and resistant strains used for insecticide resistance research are typically from two different populations, NILs can be used for avoiding the phenotypic interference from nonresistant factors found in more widely differing genetic backgrounds. Several insect NILs have been utilized for genetic analyses of insecticide resistance^28–31^, resistance gene mapping and cloning^32^, and fitness tests^33^.

In the housefly genome, a total of 92 esterase genes were predicted^34^. Given that the carboxylesterase gene *MdαE7* was suggested to be responsible for malathion resistance of houseflies^2,17^, in this manuscript we report the establishment of a NIL of malathion-resistant (N-MRS) housefly to minimize the differences in genetic backgrounds, theoretically enabling clarification of the involvement of multiple mutations and *MdαE7* overexpression in sex-specific malathion resistance. This allowed us to provide molecular evidence for a possible role of an M factor in insecticide resistance of the housefly.

## Results

### Toxicity analysis and biochemical assays

As the result of selection, compared to the susceptible CSS females, a 11-fold decrease of LD_50_ to malathion was observed in the malathion-susceptible MSS females. Meanwhile, surprisingly, the MSS males obtained 10-fold malathion resistance to the CSS males, with a significantly higher LD_50_ than the MSS females. After the process of repeated backcrossing and self-breeding under malathion selection, the near-isogenic resistant to malathion N-MRS females and males developed 56- and 54-fold resistance relative to the CSS strain, respectively (Table 1).

**Table 1:** Synergism of DEF on malathion toxicity in the MSS and N-MRS strains.

Strain	N^a^	Slope (SE)	LD_50_ (FL 95%) μg/fly	χ^2^ (df)^b^	RR^c^	SR^d^
**Females**						
CSS	289	1.1 (0.22)	9.21 (6.29–13.9)	0.61 (4)	1.0	
MSS	355	4.1 (0.80)	0.829 (0.713–0.974)	1.6 (3)	0.090	
MSS + DEF	320	6.1 (0.84)	0.445 (0.376–0.508)	5.1 (3)		1.86
N-MRS	316	5.2 (0.70)	518 (470–569)	8.8 (4)	56	
N-MRS + DEF	281	3.4 (0.39)	1.76 (1.54–2.05)	2.0 (4)		294
**Males**						
CSS	270	1.7 (0.27)	11.6 (7.38–17.6)	1.6 (3)	1.0	
MSS	412	4.8 (0.42)	119 (104–133)	2.0 (3)	10	
MSS + DEF	360	7.7 (1.8)	0.677 (0.546–0.749)	6.6 (4)		176
N-MRS	400	6.9 (0.66)	632 (585–681)	6.3 (4)	54	
N-MRS + DEF	310	1.4 (0.20)	4.96 (3.61–7.59)	9.1 (4)		127

^a^Number of houseflies used in the bioassay. ^b^Chi-square value and degrees of freedom (df) as calculated by PoloPlus. ^c^RR = LD_50_ of malathion/LD_50_ of malathion for CSS strain with corresponding genders. ^d^SR = LD_50_ of malathion/LD_50_ of malathion + DEF.

The effects of the esterase inhibitor DEF as a synergist of malathion toxicity in females and males are shown in Table 1. The synergistic ratios for the MSS males, N-MRS females, and N-MRS males pretreated with DEF were 176, 294, and 127, respectively, while the corresponding value for the MSS females was 1.86.

### Identification of mutations related to malathion resistance in *MdαE7*

The total coding region of the carboxylesterase *MdαE7* gene was successfully cloned and sequenced. Alignment of nucleotide and amino acid sequences of the cDNA fragment revealed a total of seven, non-synonymous mutations that consistently occurred in the resistant houseflies, but not the susceptible MSS females (Table 2): Ser^250^-Thr (S^250^T), Trp^251^-Ser (W^251^S), Met^303^-Ile (M^303^I), Leu^354^-Phe (L^354^F), Ser^357^-Leu (S^357^L), Trp^378^-Arg (W^378^R) and Ser^383^-Thr (S^383^T). The former six mutations were caused by single nucleotide substitutions, while in the last mutation two nucleotides (G1148 and T1149) were replaced at the Ser^383^ codon. However, a polymorphic allele at C1148 that can produce a substitution codon encoding threonine (Thr^383^) renders irrelevant the replacement of the third nucleotide in this codon because the amino acid switch only requires the G1148C substitution.

**Table 2:** Non-synonymous mutations in malathion resistant housefly^a^.

Nucleotide		Amino acid	
Site	Substitution^b^	Site	Substitution^b^
748	T → A	250	Ser → Thr
752	G → C	251	Trp → Ser
909	G → A	303	Met → Ile
1060	C → T	354	Leu → Phe
1070	C → T	357	Ser → Leu
1132	T → C	378	Trp → Arg
1148	G → C	383	Ser → Thr
1149	T → A		

^a^Mutations are generated by the corresponding nucleotide substitutions displayed in the same row. ^b^The former nucleotide or amino acid was detected in susceptible housefly and the latter in resistant housefly.

**Figure 1:**
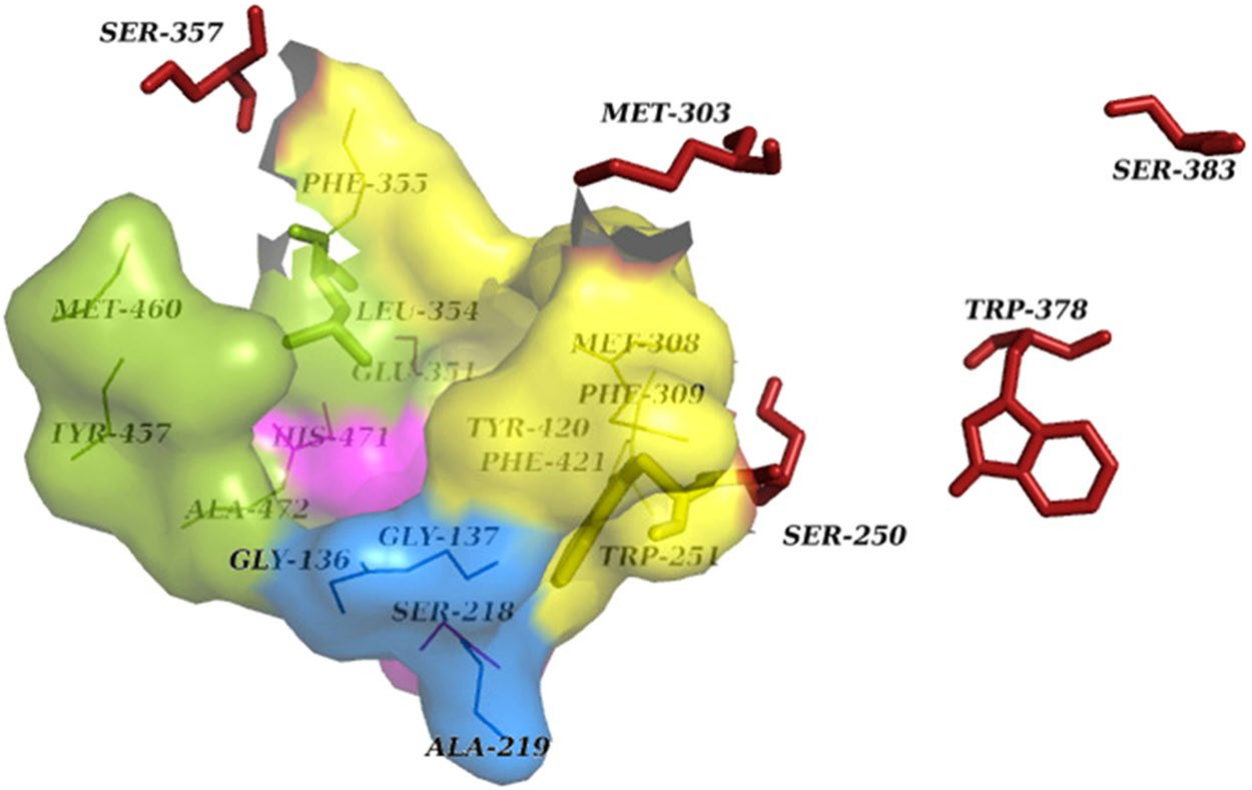
The structure of the active cavity of *MdαE7*. The catalytic triad and oxyanion hole are colored magenta and blue, respectively. The substrate binding pocket is divided into a small pocket and a large pocket, colored green and yellow, respectively. Mutations are displayed as sticks and colored dark red, except Trp^251^ and Leu^354^, which are located in the binding pocket.

Based on the three-dimensional structure of *LcαE7* (76% identical with *MdαE7*), which has been solved by X-ray crystallography, *MdαE7* gene was successfully modeled after energy minimization. Similar with *LcαE7* ^35^, the structure of active sites in *MdαE7* are comprised of a canonical, catalytic triad of the α/β-hydrolase fold (Ser^218^, His^471^ and Glu^351^), an oxyanion hole (Ala^219^, Gly^136^ and Gly^137^) and an extremely asymmetrical cavity for substrate binding. This model of the substrate binding pocket includes a small pocket (Leu^354^, Tyr^457^, Met^460^ and Ala^472^) and a large pocket (Trp^251^, Met^308^, Phe^309^, Phe^355^, Tyr^420^ and Phe^421^) (Fig. 1), revealing that two of the detected mutation sites, both Trp^251^ and Leu^354^ are in the active site region. Moreover, five of the substitutions are predicted to be close to the substrate binding cavity except for Trp^378^ and Ser^383^, which were, respectively, 21.0 and 28.0 Å away from Ser^218^, the catalytic center of the molecule.

### Correlation of polymorphic allele frequencies with malathion resistance in housefly

We conducted a point mutation screen of resistant houseflies to search for polymorphisms in 80 adult individuals from each gender of the MSS and N-MRS strains to explore their correlation with malathion resistance. We found significant differences in the occurrence of polymorphisms among different populations (Table 3). Only homozygous susceptible (wild-type) alleles of each of the seven mutation sites were carried by the MSS individuals, while homozygous mutant alleles were only detected in the N-MRS strain. Notably, homozygous susceptible alleles mainly occurred in the MSS females (96.25% to 100%) while the majority of MSS males (80% to 98.75%) were heterozygous for these alleles, showing extremely unbalanced expression of polymorphisms between genders. In contrast, consistently high frequencies of homozygous mutant alleles were examined in both the N-MRS females (86.25% to 100%) and males (93.75% to 100%).

**Table 3:** Distribution of polymorphic alleles of non-synonymous mutations in the *MdαE7*.

			Population							
			MSS−♀	MSS−♂	N-MRS- ♀	N-MRS− ♂	BC− ♀	BC− ♂	F2− ♀^b^	F2− ♂^b^
Frequency of Polymorphisms of Amino Acid Mutation Sites (%, ± SE)^a^	S250T [TCA → ACA]	T/T	97.5 ± 2.5	1.25 ± 1.25	0	0	100	1.25 ± 1.25	58.14 ± 1.76	0
		T/A	2.5 ± 2.5	98.75 ± 1.25	0	6.25 ± 3.75	0	98.75 ± 1.25	41.86 ± 1.76	51.72 ± 2.68
		A/A	0	0	100	93.75 ± 3.75	0	0	0	48.28 ± 2.68
	W251S [TGG → TCG]	G/G	98.75 ± 1.25	2.5 ± 1.44	0	0	100	1.25 ± 1.25	58.18 ± 2.05	0
		G/C	1.25 ± 1.25	97.5 ± 1.44	0	0	0	98.75 ± 1.25	41.82 ± 2.05	50.99 ± 2.60
		C/C	0	0	100	100	0	0	0	49.01 ± 2.60
	M303I [ATG → ATA]	G/G	100	20 ± 4.08	0	0	100	6.25 ± 3.75	58.46 ± 1.64	0
		G/A	0	80 ± 4.08	8.75 ± 4.27	5 ± 2.89	0	93.75 ± 3.75	41.54 ± 1.64	50.99 ± 2.60
		A/A	0	0	91.25 ± 4.27	95 ± 2.89	0	0	0	49.01 ± 2.60
	L354F [CTT → TTT]	C/C	98.75 ± 1.25	12.5 ± 3.23	0	0	100	3.75 ± 3.75	58.50 ± 1.88	0
		C/T	1.25 ± 1.25	87.5 ± 3.23	11.25 ± 2.39	5 ± 2.89	0	96.25 ± 3.75	41.50 ± 1.88	50.99 ± 2.60
		T/T	0	0	88.75 ± 2.39	95 ± 2.89	0	0	0	49.01 ± 2.60
	S357L [TCA → TTA]	C/C	100	12.5 ± 4.79	0	0	100	11.25 ± 7.18	59.55 ± 2.14	0
		C/T	0	87.5 ± 4.79	13.75 ± 2.39	6.25 ± 3.75	0	88.75 ± 7.18	40.45 ± 2.14	51.72 ± 2.68
		T/T	0	0	86.25 ± 2.39	93.75 ± 3.75	0	0	0	48.28 ± 2.68
	W378R [TGG → CGG]	T/T	96.25 ± 2.39	1.25 ± 1.25	0	0	100	0	56.72 ± 1.71	0
		T/C	3.75 ± 2.39	98.75 ± 1.25	3.75 ± 2.39	2.5 ± 1.44	0	100	43.28 ± 1.71	50.99 ± 2.60
		C/C	0	0	96.25 ± 2.39	97.5 ± 1.44	0	0	0	49.01 ± 2.60
		G/G	96.25 ± 2.39	1.25 ± 1.25	0	0	100	0	58.14 ± 1.76	0
	S383T [AGT → ACA]	G/C	3.75 ± 2.39	98.75 ± 1.25	3.75 ± 2.39	5 ± 2.89	0	100	41.86 ± 1.76	50.99 ± 2.60
		C/C	0	0	96.25 ± 2.39	95 ± 2.89	0	0	0	49.01 ± 2.60
		T/T	100	7.5 ± 3.23	0	0	100	6.25 ± 3.75	58.83 ± 1.75	0
		T/A	0	92.5 ± 3.23	3.75 ± 2.39	2.5 ± 1.44	0	93.75 ± 3.75	41.17 ± 1.75	50.99 ± 2.60
		A/A	0	0	96.25 ± 2.39	97.5 ± 1.44	0	0	0	49.01 ± 2.60

^a^Values represent mean ± SE for four replications of frequency (%) analyses of each mutation; the nucleotide polymorphisms are underlined. ^b^Data was synthesized from the four malathion-treated groups in corresponding gender.

**Figure 2:**
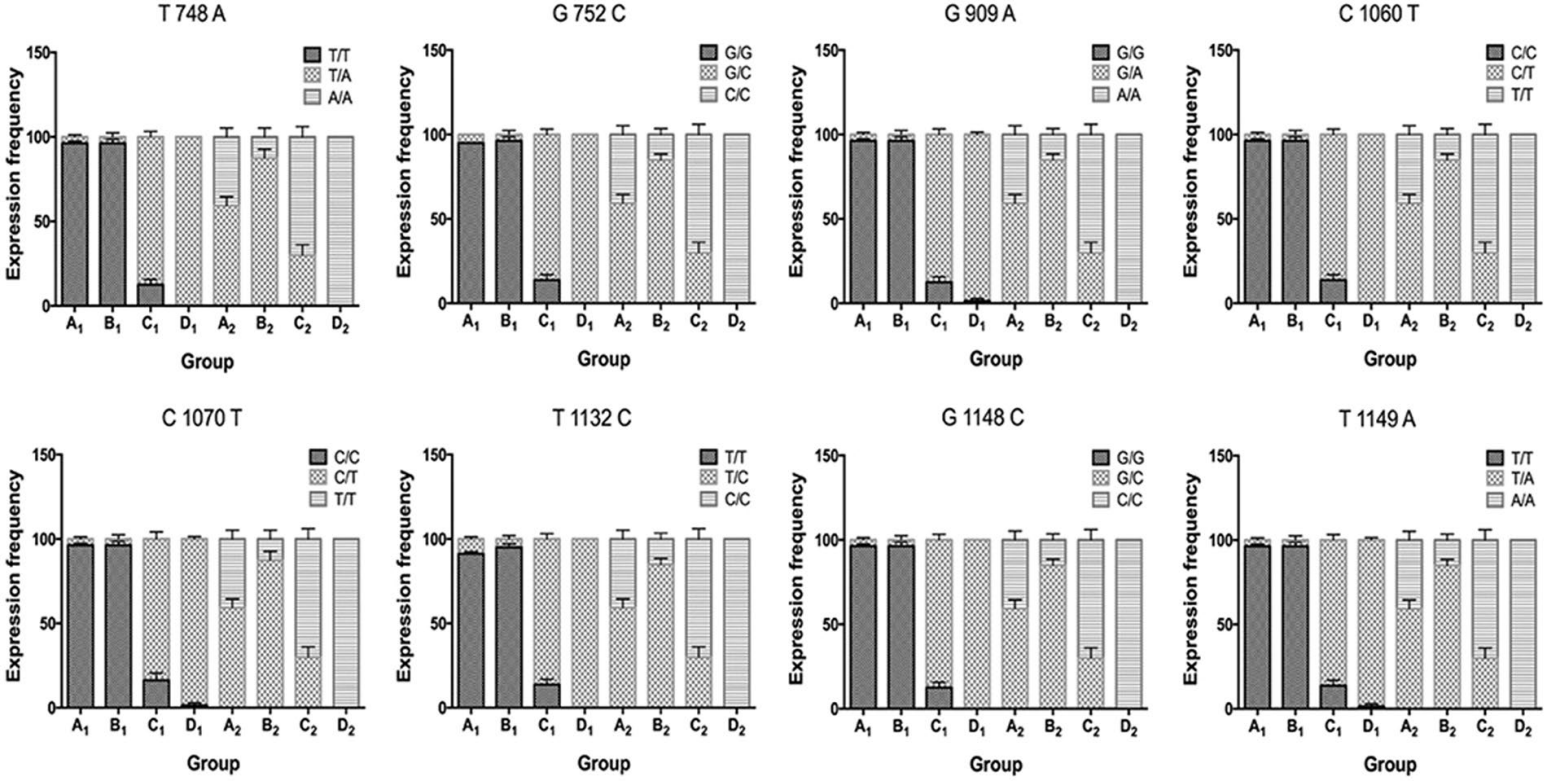
SNP mutation allele frequency distribution for each F_2_ generation housefly group, distinguished by gender and level of malathion tolerance. The frequency of allele expression shown along the Y axis is the percentage of houseflies carrying the corresponding homozygous or heterozygous allele(s). Housefly groups are shown along the X axis; A_1_, B_1_, C_1_ and D_1_ represent the groups in F_2_ females that were dead under LD_10_, between LD_10_ and LD_50_, between LD_50_ and LD_90_ and alive above LD_90_ dosage treatment, respectively; and A_2_, B_2_, C_2_ and D_2_ represent the groups in F_2_ males that were dead under LD_10_, between LD_10_ and LD_50_, between LD_50_ and LD_90_ and alive above LD_90_ dosage treatment, respectively. Error bars represent standard errors of the means (n = 4 independent replicates of treatment).

We separately examined the prevalence of each mutation in the F_2_ females and males in order to further evaluate the relationship between the polymorphic allele frequencies and increased levels of resistance to malathion. Four-day-old houseflies were treated with different concentrations of malathion and assembled into four groups (A to D), for each gender, based on their similar levels of malathion resistance (low to high, respectively) (Table 4). The results of SNP detection revealed that the frequencies of polymorphic expression at each codon site were strongly correlated with the levels of resistance in housefly (Fig. 2). High expression of homozygous susceptible alleles and low expression of heterozygous alleles in the F_2_ females were observed in groups A_1_ and B_1_, with relatively low malathion resistance. However, a significantly decreased frequency of homozygous susceptible alleles and increased frequency of heterozygous alleles was detected in group C_1_ and D_1_, accompanied by an increase in malathion resistance. Among the F_2_ males, heterozygotes were only found in the former three groups (A_2_, B_2_, and C_2_), with significantly higher occurrence in groups A_2_ and B_2_ than group C_2_. In confirmation of our predictions, our results showed an increasing trend of malathion-tolerant individuals carrying homozygous mutant alleles, for example, comprising the majority of group D_2_.

**Table 4:** Malathion treatment of females and males in F_2_ generation.

Malathion Treatments^a^												
Population	LD_10_ Treatment				LD_50_ Treatment			LD_90_ Treatment				
	N^b^	LD_10_ μg/fly^c^	N_A_ (Proportion)^d^	Group A (collected dead houseflies)	LD_50_ μg/fly^c^	N_B_ (Proportion)^d^	Group B(collected dead houseflies)	LD_90_ μg/fly^c^	N_C_ (Proportion)^d^	Group C (collected dead houseflies)	N_D_ (Proportion)^d^	Group D (collected alive houseflies)
F_2_♀	1858	1.27	315 (0.17)	F_2_♀- < LD_10_	33.47	566 (0.30)	F_2_♀-LD_10–50_	880.46	934 (0.50)	F_2_♀-LD_50–90_	43 (0.02)	F_2_♀- > LD_90_
F_2_♂	1748	216.92	65 (0.04)	F_2_♂- < LD_10_	396.2	735 (0.42)	F_2_♂-LD_10–50_	723.81	917 (0.52)	F_2_♂-LD_50–90_	31 (0.02)	F_2_♂- > LD_90_

^a^Each treatment was performed 4 times. ^b^Number of houseflies used in LD_10_ treatment. ^c^The doses of malathion administered to these houseflies are chosen according to the toxicity analysis in the F_2_ generation (Table 5). ^d^Number of collected houseflies in each group with corresponding letter (Proportion = collected individuals of each group/ individuals used in LD_10_ treatment).

### Linkage analysis

At the seven mutations, we observed that the majority of MSS females carried homozygous susceptible alleles, while the MSS males were primarily heterozygous for these same alleles. In view of current knowledge of the M factor, one possible explanation is that the mutant allele and M factor are carried on the same chromosome in heterozygous MSS males, so only male offspring can inherit the mutant form. Additionally, rare heterozygotes of certain codon sites still present in the MSS females may be caused by recombination^36^. Therefore, a series of genetic crosses was carried out to identify whether the mutant *MdαE7* gene is also linked to Mfactor in the N-MRS males.

Offspring from the F_1_, F_2_, and BC generation were separately tested for malathion resistance (Table 5). The F_1_ hybrids showed a similar level of resistance between females and males, with a LD_50_ of 359.80 and 466.05 μg/fly, respectively. However, in the F_2_ generation, a high degree of resistance to malathion was maintained in males, while a nearly 14-fold decrease was observed in females compared with their parental houseflies. The BC females and males also expressed remarkably different levels of resistance with a resistance ratio of 0.18- and 36-fold compared with the CSS strain, respectively.

**Table 5:** Malathion toxicity in the F_1_, F_2_, and BC generations.

Strain	N^a^	Slope (SE)	LD_50_ (FL 95%) μg/fly	χ^2^ (df)^b^	RR^c^
**Females**					
F_1_ (MSS♀ × N-MRS♂)	359	7.4 (0.83)	360 (331–384)	1.2 (3)	39
F_2_ (F_1_♀ × F_1_♂)	350	0.93 (0.12)	25.2 (14.6–40.7)	2.7 (3)	2.7
BC (MSS♀ × F_1_♂)	283	2.0 (0.36)	1.62 (1.23–2.47)	2.3 (3)	0.18
**Males**					
F_1_ (MSS♀ × N-MRS♂)	343	5.5 (0.67)	466 (410–514)	8.3 (4)	40
F_2_ (F_1_♀ × F_1_♂)	326	4.7 (0.68)	415 (364–479)	0.98 (3)	36
BC (MSS♀ × F_1_♂)	314	5.7 (1.0)	413 (353–469)	1.9 (3)	36

^a^Number of houseflies used in the bioassay. ^b^Chi-square value and degrees of freedom (df) as calculated by PoloPlus. ^c^RR = LD_50_ of malathion/ LD_50_ of malathion for CSS strain of corresponding gender (Table 1).

To validate our hypothesis of a linkage between the *MdαE7* gene and Mfactor in the N-MRS males at a molecular level, we examined the frequency of polymorphic expression at the seven codon sites in the F_2_ and BC generation (Table 3). Among the seven mutation sites found in F_2_ females, 56.72% to 59.55% of individuals had the homozygous susceptible alleles and 40.45% to 43.28% of individuals had heterozygous alleles. However, only heterozygous and homozygous mutant alleles were expressed in the F_2_ males, with a frequency of 50.99% to 51.72% and 48.28% to 49.01%, respectively. As for the BC offspring, no nucleotide substitutions at the seven codon sites were detected in any females, and individuals carrying heterozygous alleles were, for the most part of males, notably comprising the entire group with mutations at codon sites 378 and 383.

**Figure 3:**
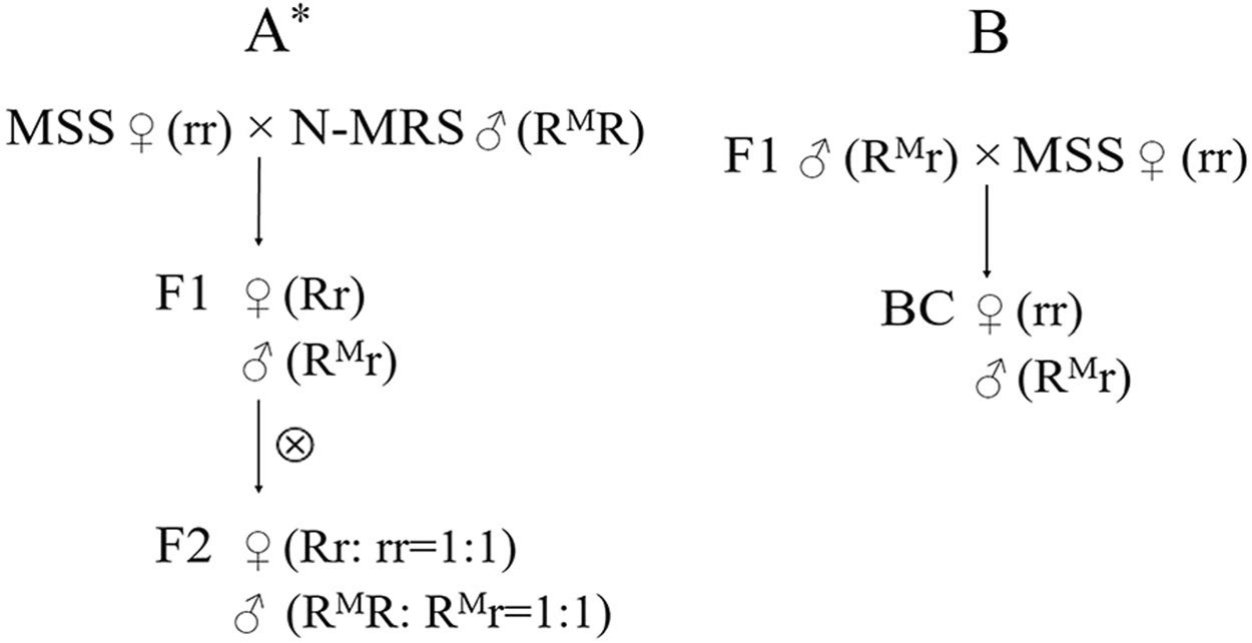
Illustration of genetic crosses with corresponding genotypes and theoretical segregation ratios. (**A**) Cross of N-MRS males with MSS females and subsequent self-cross of the F_1_ hybrids. (**B**) Backcross of F_1_ males with MSS females. *Refer to text for explanation of theoretical segregation ratios.

Since the inheritance mode of malathion resistance in housefly has been demonstrated as single, autosomal and incompletely dominant^3,37^, according to our hypothesis mentioned above, the F_1_ offspring should be all heterozygotes in both genders, regardless if the linkage exists or not (Fig. 3A). Following the law of linkage, a 1:1 segregation ratio of homozygous susceptible to heterozygous alleles is expected in F_2_ females with an intermediate level of malathion tolerance relative to MSS and F_1_ females, which carried homozygous susceptible and heterozygous alleles, respectively. As for F_2_ males, heterozygous individuals were expected to segregate in equal number to homozygous mutants, resulting in their intermediate resistance between the MSS males and the N-MRS strain. Meanwhile, BC females and males are expected to carry only homozygous susceptible and heterozygous alleles, respectively (Fig. 3B), resulting in a BC population of susceptible females and males with similar resistance to that of F_1_ males. As described above, the results of both toxicity analysis and SNP detection are relatively consistent with our assumption. However, the higher-than-expected segregation ratio (nearly 6:4) in the F_2_ females is possibly attributable to the use of only 43 individuals from the F_2_♀- > LD_90_ group for SNP detection. The unexpected expression of homozygous susceptible alleles in the BC males may be owing to rare recombination^36^ or rare heterozygotes in the N-MRS males.

### Analysis of the *MdαE7* gene expression

Comparisons of *MdαE7* gene expression were made from two aspects: expression between genders of the same strain and expression between the MSS and N-MRS strains of the same gender (Table 6). The N-MRS strain showed significantly higher mRNA expression than the MSS strain in each gender, with a p-value of 0.004 and 0.035 for females and males, respectively. However, the DNA expression did not differ between strains with both genders. In addition, no significant differences of DNA and mRNA expressions were observed between genders in both strains.

**Table 6:** Relative expression of *MdαE7* gene in the MSS and N-MRS strains.

Strain	*MdαE7*/GAPDH (Mean ± SE)		p-value between MSS and N-MRS (2-tailed)		p-value between genders (2-tailed)	
	DNA	mRNA	DNA	mRNA	DNA	mRNA
**Females**						
MSS	4.25 ± 0.751	0.164 ± 0.0269	0.164	0.004	0.115	0.590
N-MRS	6.26 ± 1.08	1.23 ± 0.265	0.164	0.004	0.084	0.132
**Males**						
MSS	6.27 ± 0.827	0.186 ± 0.0294	0.057	0.035	0.115	0.590
N-MRS	8.34 ± 0.404	2.53 ± 0.863	0.057	0.035	0.084	0.132

p-values for all two-sample comparisons were obtained by a Student’s *t*-test. Significant p-values (α < 0.05) are underlined.

## Discussion

Near-isogenic lines are important material for the study of insecticide resistance. With the aid of a rotating set of genetic methods and concurrent malathion selection, we introduced purified malathion-resistant gene(s) into the genetic background of the susceptible MSS strain without loss, to generate the resistant N-MRS strain for further study. Our data from synergistic bioassays confirmed the involvement of esterase in sex-specific resistance to malathion.

Our investigation unveiled seven, non-synonymous SNP mutations in the *MdαE7* gene in the malathion resistant populations. Several studies have reported the existence and crucial contribution of the W^251^S non-synonymous mutation in malathion resistant houseflies. It has been proposed that the replacement of W^251^ with smaller residues creates more space to accommodate substrates with bulkier acid moieties, and reduces the steric obstruction to the inversion that must occur around phosphorus during hydrolysis of OP^38–41^. Additionally, another four mutations, S^250^T, M^303^I, S^357^L and S^383^T were detected in certain housefly strains with high malathion resistance^2^. Notably, two substitutions in our study, L^354^F and W^378^R, were the first discovery of these mutations in malathion resistant houseflies. In this case, L^354^F likely coincides with the carboxylesterase binding, which is the structural equivalent of the anionic site in cholinesterase, providing the π electrons to accommodate the quaternary nitrogen of acetylcholine^42^. Kinetic assays have shown that substitution by insertion of a small aliphatic side chain resulted in a greater increase of esterase activity than that with a larger aromatic side chain in the residue F^354^ of *LcαE7* ^41^, which was surprising, given our detection of the L^354^F mutation in *MdαE7*. Since all seven mutations have also been documented in houseflies resistant to pyrethroid insecticides^43^, it has been suggested that carboxylesterase evolution via qualitative mechanisms may cause broad insecticide resistance in insects.

A strategy of comparing polymorphic codon usage bias in individuals between different populations and groups allowed us to supply proof of the potential association between each mutation and malathion resistance in housefly. Among all the mutations, a clear shift of polymorphic distribution was detected from those with primarily homozygous susceptible alleles, through those with mostly heterozygous alleles, to those with all, or nearly all, homozygous mutant alleles, correlating with increasing resistance to malathion, and ultimately supported our predictions. Future studies will examine the effects of individual or combinations of mutations on the degradation or sequestration of malathion and other insecticides.

Genetic crosses conducted for this study, in combination with bioassays and SNP detection, identified the coexistence of an M factor and mutant *MdαE7* gene on the same chromosome in both MSS and N-MRS males. Notably, this linkage in MSS males also offered a compelling explanation for their surprisingly higher level of resistance than the susceptible MSS females. To further previous research that focused on the linkage between an autosomal M factor and malathion resistance factors^23^, our study is the first report on the molecular characterization of this linkage in both the susceptible strain and the resistant near-isogenic line, thus providing valuable insights into the important role the M factor may play in sex-associated insecticide resistance of housefly.

In our work, we found that overproduction of *MdαE7* gene transcripts in the N-MRS strain indicated that the increased expression of the carboxylesterase gene was associated with malathion resistance in housefly. Similar to a resistance study in other insect species^44^, the combination of quantitative and qualitative changes in carboxylesterase may further contribute to the malathion resistance of insects. Moreover, our analysis suggested that up-regulation of the *MdαE7* gene was mainly responsible for the overabundance of the gene product. Many types of regulatory mutations can lead to changes in gene expression, and can occur either in *cis* (disruption or deletion of an upstream regulatory element in the promoter region of the gene) or in *trans* (disruption of a gene coding for a transcription factor that binds the above-mentioned *cis*-elements)^45^. Although we have obtained evidence for both *cis*- and *trans*-regulation of metabolic resistance involving glutathione *S*-transferases’ and cytochrome P450 monooxygenases’ activity, the regulatory mechanisms of esterase-mediated resistance are still poorly understood^10^. In our estimation, it is noteworthy that *MdαE7* was 2.1-fold higher expressed in males than females in the N-MRS strain, in spite of no significant difference between them. Given the linkage between M factor and *MdαE7* gene in the N-MRS males, it is reasonable to consider the possible involvement of an M factor in the *cis*-regulation of esterase genes, suggesting a novel direction for further investigation into the regulatory factors of gene overexpression.

## Methods

### Insects

Three housefly strains were used in this study.

The CSS strain, a susceptible strain derived from National Taiwan University in 1987, has been reared in laboratory without exposure to any insecticides. Malathion toxicity in this strain was included to evaluate resistance levels in each population tested in our study.

The MSS strain was selected from the CSS strain. Unmated houseflies less than 3 h old from the CSS strain were collected in this selection. Single pairs of houseflies were reared and crossed individually. Based on a previous bioassay of CSS females, partial progeny of each pair were treated with an appropriate dosage of malathion, and the most susceptible pair was selected according to the highest mortality after 24 h. Progeny without malathion treatment from the selected pair were chosen for further breeding. The MSS strain was produced after two generations with this selection.

The near-isogenic line to malathion resistance (N-MRS) strain was established using the protocols of Shan^31^, with some modifications. A wild housefly strain (CFD), originally collected from a garbage dump in Beijing, China, in 1998, and maintained in the laboratory without exposure to insecticides, showed high resistance to malathion. The CFD males and unmated MSS females were collected 3 h after emergence and crossed to produce a hybrid F_1_ population. An F_2_ population was derived from the self-breeding of F_1_ flies. The bioassay was conducted on four-day-old F_2_ females, while the rest of F_2_ population (collected within 24 h of emergence) was treated with a proper dosage of malathion to kill all the susceptible ones and most of the heterozygotes. The survivors produced an F_3_ generation. F_3_ males were backcrossed to virgin MSS females, both collected within 3 h of emergence, to produce the BC_1_ population. Subsequently, the BC_1_F_1_ population was derived from the self-breeding of BC_1_, and the same treatment for F_2_ was repeated on BC_1_F_1_. The N-MRS strain was thus constructed after 7 repeated processes of backcrossing and self-breeding with malathion selection.

### Bioassay and analysis for synergism

Topical applications were performed by delivery of a 1.1-μL drop of insecticide (in acetone) onto the thoracic notum of four-day-old adult houseflies^46^. Malathion was dissolved in acetone to produce 5–7 concentrations that gave a mortality rate greater than 0 and less than 100%. Control groups were treated with a 1.1-μL drop of acetone alone. Each of the three replicates consisted of 15 to 20 flies per dose. Treated houseflies were placed in 240 ml plastic jars with a piece of sponge saturated in sugar water. Mortality was assessed after 24 h. Flies that did not move normally were scored as dead. All tests were performed at 25 ± 1 °C.

Synergistic assays were applied to study the biochemical mechanism of resistance. Both females and males were separately tested by using *S, S, S*-tributylphosphorotrithioate (DEF), an esterase inhibitor^47^. DEF was dissolved in acetone and applied by topical application at a dose of 0.98 μg/fly 1 h before the malathion treatment. The dosage of DEF was selected experimentally as the highest dose with no control mortality.

### Sequence analysis and protein modeling of *MdαE7*

Total RNA was extracted from a pool of adult houseflies of each gender, from the MSS and N-MRS strains, with a TRIzol kit (Invitrogen, Carlsbad, CA), following the manufacturer’s instructions. The first-strand cDNA was synthesized using PrimeScript^TM^ RT Reagent kit with gDNA Eraser (Takara Biotechnology, Dalian, China). To screen for putative mutations related to resistance, the full length of *MdαE7* cDNA was amplified by using primers AE7.30 (5′ATGAATTTCAAAGTTAGTCAA3′) and AE7.11 (5′AAACAATTCCTTCTTTTTATCGA3′)^17^. Amplification started with an initial denaturation step of 94 °C for 5 min, followed by 35 cycles of PCR reaction (94 °C for 30 sec, 48 °C for 30 sec, and 72 °C for 2 min) and a final extension of 72 °C for 10 min. Purified PCR products were cloned into the pMD 18-T vector and sequenced by Beijing Genomics Institute (BGI). Sequence analyses of *MdαE7* cDNA fragments were repeated three times for each housefly sample, with different preparations of mRNA, and three TA clones sequenced from each replicate. Alignment was performed using DNAman (Lynnon Biosof, USA).

The deduced amino acid sequence of the *MdαE7* gene was modeled against the 3D structure of the *LcαE7* gene (PDB accession no.5ikx)^48^ using the Swiss-model homology modeling server^49–51^, refined using Dokholyan Lab’s Chiron Server^52^, checked and validated using Structural Analysis and Verification Server from UCLA (http://services.mbi.ucla.edu/SAVES/) and viewed using Pymol Viewer.

### Genetic cross and malathion treatment

To identify the potential linkage between the M factor and malathion resistance in *Musca domestica*, MSS females and N-MRS males within 12 h of emergence were put into a cage for mass mating to produce the hybrid F_1_. A backcross by F_1_ males to virgin MSS females, as well as a self-cross of the F_1_ generation, were subsequently conducted, and the offspring were referred to as BC and F_2_ progeny, respectively. Bioassays were performed on both genders from each generation.

Based on the bioassay results of the F_2_ generation, a corresponding dose range of LD_10_, LD_50_ and LD_90_ were obtained and utilized to generate 4 groups divided by different levels of malathion resistance in each gender. According to the previous method^53^, four-day-old F_2_ females and males were separately treated with malathion at their respective dose of LD_10_. Eight hours after treatment, the dead houseflies of each gender were collected and designated as group A (i.e., F_2_♀- < LD_10_, or F_2_♂- < LD_10_). The survivors of the LD_10_ dose were then exposed to malathion at the dose of LD_50_. Eight hours later, the dead houseflies of both genders were collected and designated group B (i.e., F_2_♀-LD_10−50_, or F_2_♂-LD_10−50_), and live flies were continually exposed to a malathion LD_90_ dose. Eight hours after treatment, the dead and surviving houseflies were separately collected and designated as group C (i.e., F_2_♀-LD_50−90_, or F_2_♂-LD_50−90_) and group D (i.e., F_2_♀- > LD_90_, or F_2_♂- > LD_90_), respectively. Each treatment was repeated 4 times, and a total of 1858 females and 1748 males were treated in this experiment.

### Nucleotide polymorphism (SNP) detection in *MdαE7*

Houseflies collected from the previous cross and malathion treatment were used in SNP determination. Four replications were conducted and a total of 80 individual houseflies from each population were utilized, with 20 for each replication. A total of 60, 31 and 43 houseflies were collected in the groups of F_2_♂- < LD_10_, F_2_♂- > LD_90_ and F_2_♀- > LD_90_, respectively, and all houseflies from the three groups were used for SNP detection. Extraction of total RNA and synthesis of the first strand cDNAs from each individual housefly were described above. Primer pair 1 (forward: 5′CGGCGAAGCAAATCGTAACT3′; reverse: 5′AAGCGATGCATGGGGAAGAG3′) was designed to amplify the specific fragment from each of the individual houseflies on which the polymorphisms reside. Amplification began with an initial step of 5-min denaturation at 94 °C. The subsequent cycling conditions consisted of denaturation at 94 °C for 30 sec, annealing at 55 °C for 30 sec, and extension at 72 °C for 1 min for 35 cycles. The final step included a 10-min extension at 72 °C. PCR products were directly sequenced and the allelic polymorphisms of each mutation were analyzed using the chromatogram viewer Chromas (Technelysium Pty Ltd). The frequencies of polymorphic expression were recorded for each mutation in all tested individuals.

### Real-time PCR

The relative transcription level and copy number of the *MdαE7* gene in females and males from the MSS and N-MRS strains were separately tested by quantitative real-time PCR. Since abdomen was reported to be the principal site of carboxylesterase activity in adult houseflies^54,55^, both genomic DNA and total RNA were extracted from abdomens of four-day-old houseflies. Modified from the method of Cao^11^, the standard curves of *MdαE7* and the internal reference gene GAPDH were generated. Briefly, primer pair 2 (forward: 5′CGCTTCCTACAATACGCTTC3′; reverse: 5′CATCGGCATGGCTTACACC3′) was designed for *MdαE7* and primer pair 3 (forward: 5′GGTCATCATCTCCGCTCCATC3′; reverse: 5′CAGTGGTGGCATGGACAGTGG3′) was for GAPDH to detect the relative expression levels of mRNA and DNA. To construct the standard plasmid, the respective fragment derived from a PCR reaction with primer pair 2 or 3 was inserted into the pMD 18-T vector. The plasmid was extracted and quantified by spectrophotometry. The plasmid mass was converted into copy concentration using the formula copies/μL = *C* × 10^−6^ × 6.02 × 10^23^/(660 × *L*), where *C* is the plasmid concentration (μg/μL) and *L* is the plasmid length (bp). The standard curve was generated using the threshold cycle of a serial 10-fold dilution of plasmid templates. PCR of 20 μL reactions were conducted using 1 μL of cDNA (equivalent to 1 μg of total RNA), or 4 μL of DNA (equivalent 0.8 μg of DNA), 10 μL of 2× SYBR *Premix Ex Taq* (Takara), 0.4 μL of each primer (10 mM), 0.4 μL of Rox (Takara), and 7.8 μL (for RNA) or 4.8 μL (for DNA) ddH_2_O. The reactions were performed on ABI PRISM 7500 HT Sequence Detection Systems, the PCR program for which consisted of an initial step at 95 °C for 2 min, followed by 40 cycles of 95 °C for 15 sec and 60 °C for 30 sec. Specificity of the amplification product was assessed by a melting curve generated by a final dissociation stage at 95 °C for 15 sec, 60 °C for 15 sec and 95 °C for 15 sec. All samples including the non-template control were run in three replicates and the experiment was independently conducted three times with different RNA and DNA preparations. The accurate initial copy number of *MdαE7* and GAPDH gene were calculated by their corresponding standard curves, respectively. The relative DNA and mRNA expressions of *MdαE7* was normalized by GAPDH (amount of *MdαE7* gene/ amount of GAPDH gene).

### Data analysis

For the bioassays, data were pooled and probit analysis was made by POLO-Plus 2.0 software (LeOra Software Lnc., Berkeley, CA)^56^. Statistical analysis of LD_50_ was based on non-overlap of 95% confidence intervals^57^. The resistance ratio (RR), the LD_50_ of the resistant population divided by the LD_50_ of the reference susceptible population, presents the level of resistance. Synergism ratios (SR) were calculated by dividing the LD_50_ of insecticide alone by the LD_50_ of insecticide with DEF.

For all two-sample comparisons, the statistically significant differences of gene expression were determined using a Student’s *t*-test (SPSS v20.0 software), with a value of p < 0.05 considered statistically significant.
